# Demographic and psychosocial predictors of major depression and generalised anxiety disorder in Australian university students

**DOI:** 10.1186/s12888-016-0961-z

**Published:** 2016-07-15

**Authors:** Louise M. Farrer, Amelia Gulliver, Kylie Bennett, Daniel B. Fassnacht, Kathleen M. Griffiths

**Affiliations:** National Institute for Mental Health Research, The Australian National University, Building 63, Eggleston Road, Canberra, ACT 2061 Australia; Young and Well Cooperative Research Centre, Abbotsford, VIC 3067 Australia; Research School of Psychology, The Australian National University, Canberra, ACT 2601 Australia

**Keywords:** Depression, GAD, University students, Risk factors, Psychosocial

## Abstract

**Background:**

Few studies have examined modifiable psychosocial risk factors for mental disorders among university students, and of these, none have employed measures that correspond to clinical diagnostic criteria. The aim of this study was to examine psychosocial and demographic risk factors for major depression and generalised anxiety disorder (GAD) in a sample of Australian university students.

**Methods:**

An anonymous web-based survey was distributed to undergraduate and postgraduate students at a mid-sized Australian university. A range of psychosocial and demographic risk factors were measured, and logistic regression models were used to examine significant predictors of major depression and GAD.

**Results:**

A total of 611 students completed the survey. The prevalence of major depression and GAD in the sample was 7.9 and 17.5 %, respectively. In terms of demographic factors, the risk of depression was higher for students in their first year of undergraduate study, and the risk of GAD was higher for female students, those who moved to attend university, and students experiencing financial stress. In terms of psychosocial factors, students with experience of body image issues and lack of confidence were at significantly greater risk of major depression, and feeling too much pressure to succeed, lack of confidence, and difficulty coping with study was significantly associated with risk of GAD.

**Conclusions:**

University students experience a range of unique psychosocial stressors that increase their risk of major depression and GAD, in addition to sociodemographic risk factors. It is important to examine psychosocial factors, as these are potentially modifiable and could be the focus of university-specific mental health interventions.

## Background

Young people attending university are at high risk of mental disorders and severe psychological distress [1, 2], and they experience these problems at higher rates than other young people their age in the population [3, 4]. The prevalence of depression is particularly high among university students [5], which can be associated with a range of immediate and future negative outcomes including poor academic performance [6] and interpersonal relationships [7], increased risk of suicide [8], and impaired performance in the workplace [9]. Coupled with the fact that numbers of students attending university are increasing in Australia [10] and internationally [11, 12], the detection, prevention, and treatment of mental health problems in this vulnerable group is an important priority.

In order to effectively target interventions to university students, there is a need to understand which students are most at risk of developing mental health problems. Several large cross-sectional studies from the United States and Australia have examined demographic correlates of depression, anxiety, and other mental disorders in university students. These studies employed large sample sizes and measures validated against clinical diagnoses. In a representative US sample, Eisenberg and colleagues found that risk of depression was higher among female postgraduate students, ethnically diverse students, and bisexual students. Women in this study were more likely to experience anxiety, and those experiencing current and previous financial stress were more likely to screen positive for both depression and anxiety [13]. Similar trends have been observed among Australian university students. Said and colleagues [14] found that risk of depression and anxiety was higher among women, low income students, homosexual and bisexual students, and those aged 25–34 years. Blanco and colleagues [15] examined risk factors for psychiatric diagnoses among US university students and found that males, those who had experienced a relationship separation or divorce, domestic students, students living in rural areas, and students living away from their parents were at greater risk of a psychiatric disorder. A number of smaller studies conducted in Australia have focused on risk factors for elevated psychological distress in university students. These studies corroborate some of the findings from the larger epidemiological studies, namely that female students, younger students, and students with financial difficulties appear to be at greater risk of experiencing moderate and severe psychological distress [3, 4].

University students are exposed to a range of unique psychosocial stressors that may increase their vulnerability to depression and anxiety. Transition to university can be a major source of stress for young adults, who may experience issues such as homesickness [16], and difficulty balancing high academic workloads with other priorities [17]. For example, a recent study by Beiter and colleagues [18] examined a range of psychosocial issues affecting university students and found that academic performance, pressure to succeed, post-graduation plans, body image, and self-esteem were among the top 10 sources of concern for students, and that experience of these issues was significantly associated with moderate to extreme stress. It is particularly important to examine the relationship between psychosocial risk factors and mental health problems in university students, given that these factors are potentially modifiable, unlike most demographic characteristics.

Examination of the literature exploring risk factors for mental disorders among university students reveals an important gap. Studies that have employed measures of depression and anxiety that correspond to clinical diagnostic criteria (such as the Patient Heath Questionnaire – 9 (PHQ-9) [19]) have focused largely on demographic risk factors. By contrast, the relatively few studies that have examined psychosocial risk factors have utilised outcome measures that measure the severity of symptomatology, such as the Kessler-10 (K-10) psychological distress scale [20] and the Depression, Anxiety, and Stress Scales – 21 (DASS-21) [21]. The current study seeks to address this gap in research on predictors of diagnostic outcomes by examining psychosocial risk factors as well as demographic risk factors for depression and anxiety among university students using measures based on the Diagnostic and Statistical Manual of Mental Disorders IV (DSM-IV) [22] criteria for major depression and generalised anxiety disorder (GAD). The study examined the following general hypotheses:

1. Demographic factors including female gender and those involving minority status or adversity (e.g. sexual or gender diversity, financial stress, relationship breakdown) will be associated with an increased risk of depression or GAD.
2. Experience of psychosocial stressors specific to university study (e.g. difficulties with time management, coping with study) will be associated with an increased risk of depression or GAD.

## Methods

### Procedure

A web-based survey of undergraduate and postgraduate students at the Australian National University (ANU) was conducted in October – December 2014. Several recruitment methods were used. First, a random sample of 5,265 undergraduate and postgraduate students was selected from the total ANU student population to receive an e-mail invitation to participate in the survey. The targeted sample comprised 33 % of the entire student population (5265/15821), and was composed of 2009 undergraduate students (22 % of the total undergraduate population), 2086 postgraduate coursework students (51 % of the total postgraduate coursework student population) and 1170 higher degree research students (48 % of the total higher degree research student population). One week after the initial invitation, a reminder e-mail was sent to those students who did not respond to the initial invitation. In addition, posters and postcards advertising the survey were distributed around the university campus, and students were encouraged to complete the survey at a mental health awareness event held on campus. The survey was also advertised on social media (Facebook) and through student focused organisations such as the ANU Student’s Association (ANUSA) and the Postgraduate and Research Student’s Association (PARSA).

Ethical approval for the survey was granted by the ANU Human Research Ethics Committee (Protocol 2014/548). Participants were required to click on a link to the survey, read an information sheet, and provide their informed consent to participate before completing the survey. In order to be eligible to participate in the survey, participants were required to confirm that they were currently enrolled as a student at the ANU and aged 18 years or older. Participants were provided with help seeking information at the end of the survey to utilise if they felt distressed, and participants who reported suicidal ideation in their survey responses were provided with contact information for crisis services.

### Survey measures

Depression was measured by the PHQ-9 [19], which assesses the frequency of DSM-IV depression symptoms over the past two weeks. PHQ-9 scores range from 0 to 27 and respondents were classified as screening positive for major depressive disorder according to the PHD-9 clinical diagnosis algorithm developed by Spitzer and colleagues [23]. According to this algorithm, respondents were classified as having major depressive disorder if they indicated having experienced five or more of the nine depressive symptoms in the PHQ-9 at least ‘more than half the days’ within a two week period, including depressed mood and/or lack of interest or pleasure. The PHQ-9 has good sensitivity (.77–.88) and specificity (.88–.94) for detecting major depression in both clinical and general population samples [24] and has been used in large epidemiological studies examining the prevalence of mental health problems in university students [13, 14]. The internal consistency of the PHQ-9 in the current sample was high (α = .89). Generalised anxiety disorder (GAD) was measured by the GAD-7, which comprises items that correspond to DSM-IV symptom criteria for GAD [25]. GAD (as opposed to other anxiety disorders) was examined in this study to enable direct comparison with other large scale prevalence studies conducted in university student populations. However, we acknowledge that the GAD-7 also predicts, and has been used as a brief screen for other anxiety conditions including social anxiety disorder, panic disorder, and PTSD [24]. Scores on the GAD-7 range from 0 to 21 and a score of ten or greater was used as a cut-off point to identify clinical cases of GAD [25]. The reliability and validity of the GAD-7 in primary care and general population samples is well supported [24, 26]. The internal consistency of the GAD-7 in the current sample was high (α = .91). The following demographic characteristics were measured: age, gender, ethnicity, country of birth, living situation, having moved to attend university, financial stress, receiving government assistance payments, relationship status, study load, status as a domestic or international student, and identification as lesbian, gay, bisexual, transgender, intersex, or queer (LGBTIQ). Psychosocial stressors were assessed by asking respondents if they had experienced any of the following while at university: feeling too much pressure to succeed, time management issues/procrastination, exam anxiety, problems achieving work/life/study balance, lack of confidence, loneliness, difficulty coping with study, body image problems, relationship issues, homesickness, and issues with sexual or gender identity.

### Statistical analysis

All analyses were conducted using SPSS Version 22 [27]. Binary logistic regression was used to examine the relationship between demographic and psychosocial predictors and presence of major depression or generalised anxiety disorder according to PHQ-9 and GAD-7 clinical cut off criteria. Demographic predictors included: age (18–25, 26–34, 35+), gender (male, female), relationship status (single, in a romantic relationship), country of birth (Australia, overseas), having moved location to attend university (moved, did not move), living situation (on campus, off campus), level of financial stress (no financial stress, occasional financial stress, frequent financial stress, constant financial stress), receiving government payments (yes, no), level of study (undergraduate, postgraduate), year of study (first year undergraduate, later year undergraduate, honours, postgraduate), study intensity (part-time, full-time), student status (international, domestic), and identification as LGBTIQ (yes, no). Experience of psychosocial predictors was coded in a binary fashion (yes, no). First, univariate associations between all predictors and major depression and GAD were investigated, and then all significant predictors were entered into a final multivariate model for major depression and GAD.

## Results

### Sample characteristics

A total of 611 students completed the survey, yielding a response rate of 11.6 % of those who were sent an e-mail invitation. Demographic and clinical characteristics of the sample are provided in Table 1. The average age of participants was 26 years (SD = 9.3, range = 18–86 years^1^), and the sample was predominantly female (62 %, *n* = 379). Compared to the overall student population at ANU, females were slightly overrepresented in the current sample (62 % versus 52 % in ANU student population), as were undergraduate students (54 % versus 48 % in the ANU student population), and students studying full-time (87 % versus 68 % in ANU student population).

**Table 1:** Demographic and clinical characteristics of the sample

	All	Undergraduates	Postgraduates
	*N* = 611	*n* = 328	*n* = 283
Age, *M (SD*)	26.26 (9.30)	21.78 (5.85)	31.46 (9.85)
Gender, *n (%)*			
Female	379 (62.0)	226 (68.9)	153 (54.1)
Male	231 (37.8)	101 (30.8)	130 (45.9)
Other	1 (0.2)	1 (0.3)	0 (0.0)
Ethnicity, *n (%)*			
Caucasian/European	375 (61.4)	228 (69.5)	147 (52.1)
Asian/Indian	199 (32.6)	87 (26.5)	112 (39.7)
Aboriginal/Torres Strait Islander/Pacific Islander	9 (1.5)	4 (1.2)	5 (1.8)
Latino/South American	8 (1.3)	1 (0.3)	7 (2.5)
African	7 (1.1)	2 (0.6)	5 (1.8)
Middle Eastern	4 (0.7)	0 (0.0)	4 (1.4)
Multiple/Other	8 (1.3)	6 (1.8)	2 (0.7)
Born in Australia	351 (57.4)	226 (68.9)	125 (44.2)
Moved to attend university	389 (63.7)	205 (62.5)	184 (65.0)
Living situation			
University residential housing	219 (35.8)	146 (44.5)	73 (25.8)
With parents	93 (15.2)	83 (25.3)	10 (3.5)
With partner and/or children	109 (17.8)	21 (6.4)	88 (31.1)
Alone	34 (5.6)	9 (2.7)	25 (8.8)
Other off campus	156 (25.5)	69 (21.0)	87 (30.7)
Financial stress			
No financial stress	187 (30.6)	80 (24.4)	107 (37.8)
Occasional financial stress	308 (50.4)	182 (55.5)	126 (44.5)
Frequent financial stress	67 (11.0)	35 (10.7)	32 (11.3)
Constant financial stress	49 (8.0)	31 (9.5)	18 (6.4)
Receiving assistance payments from Government	185 (30.3)	97 (29.6)	88 (31.1)
Relationship status			
Single	307 (50.2)	198 (60.4)	109 (38.5)
In a romantic relationship	304 (49.8)	130 (39.6)	174 (61.5)
Study load			
Full-time	530 (86.7)	310 (94.5)	220 (77.7)
Part-time	81 (13.3)	18 (5.5)	63 (23.3)
International student	162 (26.5)	44 (13.4)	118 (41.7)
Identify as LGBTIQ	52 (8.5)	36 (11.0)	16 (5.7)
PHQ-9, *M* (*SD*)	7.33 (5.73)	8.37 (6.08)	6.05 (4.97)
GAD-7, *M (SD)*	5.60 (5.04)	6.44 (5.26)	4.55 (4.56)

Postgraduate students were older (*t* (444) = -14.5, *p* < .001), more likely to be an international student (χ^2^ = 62.4, *p* < .001), and more likely to be in a romantic relationship (χ^2^ = 29.0, *p* < .001) compared to undergraduate students. Postgraduate students also reported lower depression symptoms (*t* (497) = 4.7, *p* < .001) and lower anxiety symptoms (*t* (494) = 4.2, *p* < .001) than undergraduate students.

### Prevalence of mental disorders

7.9 % of respondents (*n* = 48) met the clinical criteria for a diagnosis of major depressive disorder on the PHQ-9. 17.5 % of respondents (*n* = 107) met the clinical criteria for a diagnosis of GAD on the GAD-7.

### Demographic risk factors for major depression and GAD

Table 2 shows the univariate and multivariate associations between demographic risk factors and presence of major depression and GAD, respectively. The odds of meeting clinical criteria for major depression were lower among students in a romantic relationship. The risk of depression was higher for students who moved location to attend university, those who experience frequent financial stress, domestic students, and students in their first year of undergraduate study. In the final multivariate model, students in their first year of study remained at significantly greater risk of experiencing major depression.

**Table 2:** Demographic risk factors for major depression and GAD

Demographic risk factors	Major depression		GAD	
	Univariate	Final multivariate model	Univariate	Final multivariate model
	OR (95 % CI)		OR (95 % CI)	
Age 18–25	Reference		Reference	
Age 26–34	0.46 (0.20, 1.05)		0.53 (0.30, 0.94)*	0.81 (0.39, 1.67)
Age 35+	0.34 (0.10, 1.12)		0.68 (0.35, 1.31)	0.74 (0.33, 1.68)
Male	Reference		Reference	
Female	1.92 (0.98, 3.76)		2.71 (1.64, 4.47)**	1.94 (1.15, 3.29)*
Single	Reference		Reference	
In a romantic relationship	0.46 (0.25, 0.86)*	0.53 (0.28, 1.03)	0.95 (0.62, 1.44)	
Born overseas	Reference		Reference	
Born in Australia	1.59 (0.85, 2.95)		2.28 (1.44, 3.61)**	1.01 (0.56, 1.83)
Did not move to attend university	Reference		Reference	
Moved to attend university	2.11 (1.17, 3.80)*	1.83 (0.97, 3.43)	2.04 (1.34, 3.11)**	1.77 (1.10, 2.84)*
Living on campus	Reference		Reference	
Living off campus	1.17 (0.63, 2.17)		1.07 (0.70, 1.66)	
No financial stress	Reference		Reference	
Occasional financial stress	1.70 (0.80, 3.60)	1.58 (0.73, 4.42)	2.22 (1.25, 3.96)**	1.91 (1.05, 3.48)*
Frequent financial stress	2.75 (1.06, 7.09)*	2.42 (0.91, 6.46)	4.36 (2.07, 8.77)**	3.70 (1.74, 7.87)**
Constant financial stress	1.15 (0.31, 4.37)	1.00 (0.26, 3.87)	4.00 (1.81, 8.86)**	3.47 (1.51, 7.97)**
Not receiving government payments	Reference		Reference	
Receiving government payments	0.82 (0.42, 1.60)		1.09 (0.70, 1.71)	
Postgraduate	Reference		Reference	
First year undergraduate	4.50 (2.11, 9.58)**	2.98 (1.33, 6.69)**	2.49 (1.40, 4.45)**	1.44 (0.67, 3.05)
Later year undergraduate	1.85 (0.87, 3.93)	1.40 (0.63, 3.12)	1.88 (1.14, 3.12)*	1.34 (069, 2.61)
Honours	1.43 (0.31, 6.67)	1.30 (0.27, 6.23)	3.61 (1.56, 8.32)**	2.23 (0.86, 5.76)
Part time study	Reference		Reference	
Full time study	1.38 (0.53, 3.58)		1.59 (0.79, 3.20)	
International student	Reference		Reference	
Domestic student	3.41 (1.33, 8.76)*	2.16 (0.77, 6.01)	3.73 (1.94, 7.17)**	2.04 (0.88, 4.74)
Not LGBTIQ	Reference		Reference	
LGBTIQ	1.57 (0.63, 3.87)		1.47 (0.74, 2.90)	

*p < .05, **p < .01

The odds of GAD were lower for those aged 26–34, and higher among women, students who moved location to attend university, students born in Australia, domestic students, and students experiencing occasional, frequent, and constant financial stress. In the final multivariate model, being female, having moved to attend university, and experience of financial stress remained significantly associated with greater risk of GAD.

### Psychosocial risk factors for major depression and GAD

Table 3 shows the prevalence of psychosocial risk factors in the sample, and the univariate and multivariate associations between these factors and major depression and GAD. The most prevalent psychosocial issues experienced by students were feeling too much pressure to succeed, issues with time management and procrastination, exam anxiety, managing work/life balance, and lack of confidence. All predictors were univariately associated with increased risk of major depression and GAD, with the exception of homesickness which was not significantly associated with major depression or GAD and issues with sexual or gender identity which was not associated with GAD. In the final multivariate model, those with experience of body image issues and lack of confidence remained at significantly greater risk of major depression. Feeling too much pressure to succeed, lack of confidence, and difficulty coping with study remained significantly associated with risk of GAD in the final model.

**Table 3:** Psychosocial risk factors for major depression and GAD

Psychosocial risk factor		Major depression		GAD	
		Univariate	Final multivariate model	Univariate	Final multivariate model
	*n* (%)	OR (95 % CI)		OR (95 % CI)	
Feeling too much pressure to succeed	388 (60.9)	9.86 (3.03, 32.10)**	2.64 (0.73, 9.49)	8.04 (3.97, 16.26)**	3.44 (1.57, 7.55)**
Time management issues/procrastination	387 (60.8)	3.77 (1.67, 8.55)**	0.98 (0.37, 2.62)	3.18 (1.88, 5.38)**	1.02 (0.53, 1.96)
Exam anxiety	378 (59.3)	6.01 (2.35, 15.38)**	1.78 (0.64, 4.96)	2.45 (1.50, 3.99)**	0.74 (0.41, 1.34)
Problems achieving work/life/study balance	356 (55.9)	4.02 (1.85, 8.73)**	1.12 (0.44, 2.86)	3.12 (1.90, 5.10)**	0.97 (0.52, 1.80)
Lack of confidence	328 (51.5)	11.09 (3.94, 31.25)**	3.84 (1.28, 11.56)*	5.10 (3.01, 8.62)**	2.17 (1.17, 3.82)*
Loneliness	288 (45.2)	5.63 (2.68, 11.82)**	1.91 (0.84, 4.34)	3.56 (2.26, 5.63)**	1.44 (0.86, 2.43)
Difficulty coping with study	265 (41.6)	6.66 (3.17, 13.99)**	2.30 (0.93, 5.67)	5.42 (3.36, 8.74)**	2.77 (1.53, 5.02)**
Body image problems	218 (34.2)	4.67 (2.47, 8.77)**	2.11 (1.06, 4.20)*	2.79 (1.82, 4.28)**	1.54 (0.95, 2.49)
Relationship issues	171 (26.8)	2.06 (1.14, 3.74)*	0.88 (0.46, 1.71)	2.52 (1.63, 3.89)**	1.27 (0.78, 2.07)
Homesickness	154 (24.2)	1.34 (0.71, 2.54)		1.12 (0.71, 1.81)	
Issues with sexual or gender identity	46 (7.5)	3.75 (1.73, 8.11)**	1.82 (0.77, 4.30)	1.97 (1.00, 3.89)	

*p < .05, **p < .01

## Discussion

This is the first study to examine both demographic and psychosocial risk factors for major depression and GAD in an Australian sample of university students. A strength of the study is the online method of survey delivery, which allowed respondents to remain anonymous and thereby may have reduced social desirability bias and encouraged disclosure. It is important to examine both stable and modifiable factors that are associated with the development of mental disorders in vulnerable populations, as this knowledge can be used to inform how interventions are developed and targeted to these groups.

The prevalence of major depression in this sample was slightly higher than has been reported among US university students (5.2 %) [13] but was similar to an estimate obtained in a sample of Australian students (8 %) [14]. Prevalence of GAD was higher in the current sample compared to the estimates obtained in both US (2.9 %) [13] and Australian (12.6 %) [14] samples, but comparable to an estimate obtained in a study of community college students in the US (17.6 %) [28]. The higher rate of GAD observed in this study may potentially be due to differences in how GAD was measured. The studies by Eisenberg et al. [13] and Said et al. [14] utilised GAD items from the Prime-MD Patient Health Questionnaire [23], which is a briefer predecessor to the more recently developed GAD-7 measure utilised in the current study. Self-selection of respondents into the study with experience of mental health problems may also partially account for the slightly elevated rates of depression and GAD observed. In addition, demographic differences between the current study sample and comparison samples are possible, and as a consequence a high degree of caution is required when interpreting any potential differences. For example, the current sample had a higher proportion of female respondents (65 %) who were experiencing higher rates of GAD, which may have inflated the overall rates.

Consistent with the literature from university [13, 14] and general population [26] samples, women in the current study were significantly more likely to experience GAD than males.

Undergraduate students, particularly first year students, were more likely to experience depression than postgraduate students, which has been demonstrated previously [14]. This may reflect the significant role transition associated with commencing tertiary study and suggests that the first year of university may be a particularly vulnerable period of adjustment for students. For many students, commencing university is associated with leaving home for the first time, and increased independence, pressure, and responsibility [29]. Thus, there is a need for intervention efforts to target students undergoing transition to university, and this has been recognised by the development of induction and peer-mentoring programs targeting first year university students [30].

A consistent finding in the literature is that financial stress is a significant risk factor for depression, anxiety, and psychological distress among university students [3, 4, 13, 14], and the results of the current study support this. Experience of mild, moderate, and severe financial stress was strongly related to GAD in the current sample, which suggests that worry about money is a significant concern for university students. Many students experience increased financial independence while at university, and must contend with high living expenses and limited ability to work while managing the demands of study [31]. There is also evidence that concern about finances increases throughout the years of university, and that many students worry about the debt that they will have accrued upon leaving university [32]. This signals the need for a multi-level approach that examines systemic issues associated with government payments for students and housing affordability, as well as initiatives from within universities to offer financial assistance and support the mental health of students experiencing financial stress.

The results of the current study suggest that the presence of social support may be a key protective factor in the experience of depression and anxiety among university students. Being in a romantic relationship was associated with lower risk of depression, and those who had moved to attend university were at higher risk of depression and GAD, perhaps due to the removal of previous sources of social support. Indeed, living away from parents has been found to be associated with mental disorders in university students [15]. Initiatives designed to increase social connectedness may prevent the development of depression and anxiety among students lacking in social support.

Surprisingly, domestic students were found to be at greater risk of major depression and GAD than international students. This finding is at odds with literature suggesting that international students are particularly vulnerable to mental health problems at university, due to the challenges associated with adjusting to an unfamiliar social and academic environment, and a tendency to delay professional help seeking for mental health problems [33, 34]. It is possible that despite the anonymous nature of the survey, international students were more reluctant to disclose mental health problems than domestic students.

In contrast to the literature, we found no evidence that identification as LGBTIQ was associated with major depression or GAD. Previous studies suggest that homosexual and bisexual students are at increased risk of experiencing depression and anxiety [13, 14], and the null finding in the current study may be due to a lack of differentiation in the way sexual orientation was measured (i.e. overall identification as LGBTIQ instead of identification as homosexual, bisexual, etc.) or the small number of respondents identifying as LGBTIQ. Indeed, differences in the prevalence of mental health problems have been observed between university students who identify with different sexual or gender orientations [35]. However, experiencing issues associated with gender or sexuality was related to an increased risk of depression, suggesting that sexual and gender identity may affect the mental health of students who are unsure about their sexuality or who have experienced stigma or a lack of acceptance from others. There is evidence to suggest that uncertainty about sexual or gender identity and the experience of interpersonal or structural stigma and discrimination increases the risk of psychological distress and mental disorders among sexual and gender diverse individuals [36, 37]. Many university campuses have developed and implemented policies, campaigns, and collectives to promote safety and inclusion for gender and sexually diverse students. It is important that these initiatives not only support students who openly identify as LGBTIQ, but those who may be questioning their gender or sexuality and may feel marginalised by both the heterosexual and LGBTIQ communities.

The results suggest that numerous psychosocial factors influence the mental health of university students. Indeed, the majority of respondents indicated that they struggle with issues that are intrinsic to the experience of being a university student, such as pressure to succeed, coping with study, exam anxiety, and difficulties achieving work/life balance. This is consistent with previous studies that have found elevated psychological distress among students who struggle to adapt to the pressures of university life [18, 29]. These findings provide important clues about the types of issues that cause university students the most distress and are thus the most critical to target in interventions designed for university students. If interventions targeting mental health problems in universities address the psychosocial issues that are of most concern to students, this may promote intervention engagement and effectiveness.

Body image issues were significantly associated with increased risk of depression in the current sample. Negative body image and eating disorders are highly prevalent among university students [38], and results of the current study suggest that transdiagnostic interventions that target co-morbid conditions (e.g. body image and depression) may be of benefit to university students.

### Limitations

An important limitation to this study is response bias. It is likely that those with personal experience with mental disorders or related difficulties were more likely to participate in the survey, thereby inflating the prevalence of mental disorders found in this sample. However, previous research has reported similar findings whether the sample was weighted or unweighted for response bias [13] suggesting that the risk factor patterns found in the current study are unlikely to reflect selection bias. Finally, we acknowledge that the cross-sectional design of this survey does not allow inferences to be made about any possible causal relationships between the predictor and outcome variables.

## Conclusions

Findings from the current study can be used to inform the development of interventions to improve the mental health of university students. Help seeking for mental health problems among university students is low [39], and is largely due to factors such as privacy concerns, lack of time, and financial constraints [40, 41]. Online interventions offer promise for treating mental health problems in university students [42] because they can be accessed anonymously, at any time, and for low or no cost to the end user. Studies also suggest that university students hold favourable attitudes toward online mental health interventions, especially those that target issues specific to university study [43, 44]. Knowledge about the modifiable factors that increase risk for mental disorders among university students provides an opportunity to develop tailored interventions that offer instrumental and psychological strategies to address issues such as procrastination, perfectionism, time management, and adjustment to university life. The results also suggest that comprehensive transdiagnostic interventions that target multiple mental disorders and related issues may be suitable for university students [44–46].

## Abbreviations

ANU, Australian National University; ANUSA, Australian National University Students Association; DASS-21, depression, anxiety, and stress scales – 21; DSM-IV, diagnostic and statistical manual of mental disorders – IV; GAD, generalised anxiety disorder; GAD-7, generalised anxiety disorder – 7; K-10, Kessler 10 psychological distress scale; LGBTIQ, lesbian, gay, bisexual, transgender, intersex, or queer; PARSA, Postgraduate and Research Students Association; PHQ-9, patient health questionnaire – 9; SD, standard deviation

## Footnotes

1 A participant aged 86 was identified as an outlier. Identical results were obtained when the analyses were conducted with and without the outlier included.

## References

[1] Leahy CM, Peterson RF, Wilson IG, Newbury JW, Tonkin AL, Turnbull D (2010). Distress levels and self-reported treatment rates for medicine, law, psychology and mechanical engineering tertiary students: cross-sectional study. Aust N Z J Psychiatry.

[2] Storrie K, Ahern K, Tuckett A (2010). A systematic review: students with mental health problems--a growing problem. Int J Nurs Pract.

[3] Cvetkovski S, Reavley NJ, Jorm AF (2012). The prevalence and correlates of psychological distress in Australian tertiary students compared to their community peers. Aust N Z J Psychiatry.

[4] Stallman HM (2010). Psychological distress in university students: a comparison with general population data. Aust Psychol.

[5] Ibrahim AK, Kelly SJ, Adams CE, Glazebrook C (2013). A systematic review of studies of depression prevalence in university students. J Psychiatr Res.

[6] Hysenbegasi A, Hass SL, Rowland CR (2005). The impact of depression on the academic productivity of university students. J Ment Health Policy Econ.

[7] Salzer MS (2012). A comparative study of campus experiences of college students with mental illnesses versus a general college sample. J Am Coll Health.

[8] Garlow SJ, Rosenberg J, Moore JD, Haas AP, Koestner B, Hendin H (2008). Depression, desperation, and suicidal ideation in college students: results from the American Foundation for Suicide Prevention College Screening Project at Emory University. Depress Anxiety.

[9] Harvey SB, Glozier N, Henderson M, Allaway S, Litchfield P, Holland-Elliott K (2011). Depression and work performance: an ecological study using web-based screening. Occup Med (Lond).

[10] Department of Education and Training. 2005-2014 Student Summary Time Series. 2015, Australian Government Department of Education and Training.

[11] U.S. Department of Education. Digest of Education Statistics, 2013 (NCES 2015-011). 2015, National Center for Education Statistics.

[12] Universities UK, Higher Education Statistics Agency (2014). Patterns and trends in UK higher education.

[13] Eisenberg D, Gollust SE, Golberstein E, Hefner JL (2007). Prevalence and correlates of depression, anxiety, and suicidality among university students. Am J Orthopsychiatry.

[14] Said D, Kypri K, Bowman J (2013). Risk factors for mental disorder among university students in Australia: findings from a web-based cross-sectional survey. Soc Psychiatry Psychiatr Epidemiol.

[15] Blanco C, Okuda M, Wright C, Hasin DS, Grant BF, Liu SM (2008). Mental health of college students and their non-college-attending peers: results from the National epidemiologic study on alcohol and related conditions. Arch Gen Psychiatry.

[16] Thurber CA, Walton EA (2012). Homesickness and adjustment in university students. J Am Coll Health.

[17] Ross SE, Niebling BC, Heckert TM (1999). Sources of stress among college students. Soc Psychol.

[18] Beiter R, Nash R, McCrady M, Rhoades D, Linscomb M, Clarahan M (2015). The prevalence and correlates of depression, anxiety, and stress in a sample of college students. J Affect Disord.

[19] Kroenke K, Spitzer RL, Williams JB (2001). The PHQ-9: validity of a brief depression severity measure. J Gen Intern Med.

[20] Kessler RC, Andrews G, Colpe LJ, Hiripi E, Mroczek DK, Normand SL (2002). Short screening scales to monitor population prevalences and trends in non-specific psychological distress. Psychol Med.

[21] Lovibond SH, Lovibond PF (2004). Manual for the depression anxiety stress scales.

[22] American Psychiatric Association (1994). Diagnostic and statistical manual of mental disorders.

[23] Spitzer RL, Kroenke K, Williams JB (1999). Validation and utility of a self-report version of PRIME-MD: the PHQ primary care study. Primary care evaluation of mental disorders. Patient health questionnaire. JAMA.

[24] Kroenke K, Spitzer RL, Williams JB, Lowe B (2010). The patient health questionnaire somatic, anxiety, and depressive symptom scales: a systematic review. Gen Hosp Psychiatry.

[25] Spitzer RL, Kroenke K, Williams JB, Lowe B (2006). A brief measure for assessing generalized anxiety disorder: the GAD-7. Arch Intern Med.

[26] Lowe B, Decker O, Muller S, Brahler E, Schellberg D, Herzog W (2008). Validation and standardization of the Generalized Anxiety Disorder Screener (GAD-7) in the general population. Med Care.

[27] IBM Corp (2013). IBM SPSS statistics for windows, version 22.0.

[28] Fortney JC, Curran GM, Hunt JB, Cheney AM, Lu L, Valenstein M (2016). Prevalence of probable mental disorders and help-seeking behaviors among veteran and non-veteran community college students. Gen Hosp Psychiatry.

[29] Verger P, Combes JB, Kovess-Masfety V, Choquet M, Guagliardo V, Rouillon F (2009). Psychological distress in first year university students: socioeconomic and academic stressors, mastery and social support in young men and women. Soc Psychiatry Psychiatr Epidemiol.

[30] Tate S, Hopkins P (2013). Re-thinking undergraduate students’ transitions to, through and out of university.

[31] Devlin M, James R, Grigg G (2008). Studying and working: a national study of student finances and student engagement. Tert Educ Manag.

[32] Cooke R, Barkham M, Audin K, Bradley M, Davy J (2004). Student debt and its relation to student mental health. J Furth High Educ.

[33] Forbes-Mewett H, Sawyer A-M (2011). Mental health issues amongst International students in Australia: perspectives from professionals at the coal-face in the Australian sociological association conference local lives/global networks.

[34] Bradley G (2000). Responding effectively to the mental health needs of international students. High Educ.

[35] Przedworski JM, VanKim NA, Eisenberg ME, McAlpine DD, Lust KA, Laska MN (2015). Self-reported mental disorders and distress by sexual orientation: results of the Minnesota college student health survey. Am J Prev Med.

[36] Mays VM, Cochran SD (2001). Mental health correlates of perceived discrimination among lesbian, gay, and bisexual adults in the United States. Am J Public Health.

[37] Oswalt SB, Wyatt TJ (2011). Sexual orientation and differences in mental health, stress, and academic performance in a national sample of U.S. college students. J Homosex.

[38] Klemchuk HP, Hutchinson CB, Frank RI (1990). Body dissatisfaction and eating-related problems on the college campus: usefulness of the Eating Disorder Inventory with a nonclinical population. J Couns Psychol.

[39] Eisenberg D, Golberstein E, Gollust SE (2007). Help-seeking and access to mental health care in a university student population. Med Care.

[40] Hunt J, Eisenberg D (2010). Mental health problems and help-seeking behavior among college students. J Adolesc Health.

[41] Givens JL, Tjia J (2002). Depressed medical students’ use of mental health services and barriers to use. Acad Med.

[42] Farrer L, Gulliver A, Chan JK, Batterham PJ, Reynolds J, Calear A (2013). Technology-based interventions for mental health in tertiary students: systematic review. J Med Internet Res.

[43] Ryan ML, Shochet IM, Stallman HM (2010). Universal online interventions might engage psychologically distressed university students who are unlikely to seek formal help. Adv Ment Health.

[44] Farrer L, Gulliver A, Chan JK, Bennett K, Griffiths KM (2015). A virtual mental health clinic for University students: a qualitative study of end-user service needs and priorities. JMIR Ment Health.

[45] Musiat P, Conrod P, Treasure J, Tylee A, Williams C, Schmidt U (2014). Targeted prevention of common mental health disorders in university students: randomised controlled trial of a transdiagnostic trait-focused web-based intervention. PLoS One.

[46] Mullin A, Dear BF, Karin E, Wootton BM, Staples LG, Johnston L (2015). The UniWellbeing course: A randomised controlled trial of a transdiagnostic internet delivered cognitive behavioural therapy (CBT) programme for university students with symptoms of anxiety and depression. Internet Interv.

